# Electrochemical properties of novel FeV_2_O_4_ as an anode for Na-ion batteries

**DOI:** 10.1038/s41598-018-27083-z

**Published:** 2018-06-11

**Authors:** Irish Valerie B. Maggay, Lyn Marie Z. De Juan, Jeng-Shin Lu, Mai Thanh Nguyen, Tetsu Yonezawa, Ting-Shan Chan, Wei-Ren Liu

**Affiliations:** 1 Department of Chemical Engineering, Chung Yuan Christian University, Taoyuan City, Chungli, 32023 Taiwan; 2 Division of Materials Science and Engineering, Faculty of Engineering, Hokkaido University Kita 13 Nishi 8, Kita-ku, Sapporo, Hokkaido, 060-8628 Japan; 3 National Synchrotron Radiation Research Center (NSRRC), 30076 Hsinchu, Taiwan

## Abstract

Spinel based transition metal oxide – FeV_2_O_4_ is applied as a novel anode for sodium-ion battery. The electrochemical tests indicate that FeV_2_O_4_ is generally controlled by pseudo-capacitive process. Using cost-effective and eco-friendly aqueous based binders, Sodium-Carboxymethylcellulose/Styrene butadiene rubber, a highly stable capacity of ~97 mAh∙g^−1^ is obtained after 200 cycles. This is attributed to the strong hydrogen bonding of carboxyl and hydroxyl groups indicating superior binding with the active material and current collector which is confirmed by the ***ex-situ*** cross-section images of the electrode. Meanwhile, only ~27 mAh∙g^−1^ is provided by the electrode using poly(vinylidene difluoride) due to severe detachment of the electrode material from the Cu foil after 200 cycles. The obtained results provide an insight into the possible applications of FeV_2_O_4_ as an anode material and the use of water-based binders to obtain highly stable electrochemical tests for sodium-ion battery.

## Introduction

The commercialization of Lithium ion batteries (LIBs) by Sony in 1991, paved the way for the development of portable devices^1^. However, the excessive costs and geographical constraints of lithium resources, made it impossible for LIBs to sustain and meet the growing demands of rechargeable batteries^2–12^. As a result, alternative battery systems are being explored. One of the most notable systems is the Sodium ion batteries (SIBs) owing to its abundance, low cost and availability^8,11,13–15^. Na atom is larger and heavier than Li atom (1.02 Å vs. 0.76 Å)^16,17^; therefore, the gravimetric and volumetric energy densities of SIBs are generally lower than LIBs. Nevertheless, energy density would not pose a great issue in terms of large-scale energy storage systems^9,12,18^. Numerous progresses on SIBs greatly focus on cathode materials, and although there is a growing number of studies on anode materials, most studies are limited to hard carbons^19–21^. Hard carbon, a non-graphitic carbonaceous compound, is given the highest considerations due to its large interlayer distance disorder structure^3,22^. Pyrolized glucose derived hard carbon prepared by Stevens and Dahn delivered a reversible sodium capacity of 300 mAh∙g^−1^ ^22,23^ However, non-graphitic carbonaceous compounds suffer from high irreversible capacity loss and low capacity retention^22^. Na-alloying type anodes, such as Sn, Sb, P, Ge and In have also been reported to deliver high reversible capacities^22,24,25^. Howbeit, these materials suffer from large volume change during electrochemical tests which results in electrode pulverization, loss of contact with the current collector, and subsequent capacity fading^24,26^.

Transition metal oxides (TMOs) have been greatly studied on LIBs due to their high theoretical capacities (>600 mAh·g^−1^) which is obtained through conversion reaction of oxides with Li^12,21,27–30^. The reduction of metal ions during the lithiation process leads to higher capacities than the commercial graphite anode (in LIBs)^21^. Furthermore, in conversion based anode materials, the anode materials are converted and new phases are expected to form^13^. TMOs can store energy through the conversion of the metals (reduction and oxidation), as well as alloying and de-alloying, which provides high reversibility. Ternary TMOs are known to provide higher electronic conductivities than simple metal oxides^31^. The first conversion anode material for SIBs was introduced by Alcantara’s group^32^. When they discharged the battery from 4.1 to 0.0 V, it delivered an initial capacity ~350 mAh·g^−1^ and subsequently decreased to ~250 mAh·g^−1^ at 0.2 V after four cycles. It is generally lower than that of LIBs, but in comparison to hard and soft carbon, NiCo_2_O_4_ still provided a higher capacity.

Vanadium metal takes multiform valence states which can provide series of transition metal vanadium-based compounds (A_x_V_y_B_z_) (A=Co, Cr, Fe, Zn, Mn, Mg, Bi, etc., B=O, S or Se)^33^. These vanadium-based compounds have been widely used as electrodes for rechargeable batteries for more than 30 years^34^. In LIB systems, numerous vanadium containing compounds have been studied such as, ZnV_2_O_4_^35,36^, CoV_2_O_6_^37^, CuV_2_O_6_^38^, Cu_2.33_V_4_O_11_^39^, FeVO_4_^33,40^ and so forth. However, several of these compounds have not been yet applied to Na-ion battery systems. It is known that at 1.0 V, it can achieve multi-electron transfer due to its multivalent properties, indicating a possibility of higher capacity delivery than Ti-based anodes^34,41^. Iron-based materials are known to provide high theoretical capacity due to its multi-valence states (Fe^0^, Fe^2+^, and Fe^3+^) which provide redox pairs of Fe^0^/Fe^2+^, Fe_0_/Fe^3+^ and Fe^2+^/Fe^3+^. Furthermore, iron is a highly abundant element with a comparable price to commercial activated carbon, hence making it economically viable for industrial-scale applications^42^. With these advantages provided by iron and vanadium oxides, Fe-V-O compound is expected to exhibit notable electrochemical performance for SIBs applications. Based on these ideas, spinel oxide FeV_2_O_4_ was prepared and its electrochemical performance was analyzed in this study. The magnetic, orbital and structure phase transitions of FeV_2_O_4_^43–45^ have been widely investigated but it has not been employed as an anode material for both Li and Na-ion battery systems.

The effects of different binders were also carried out in this study. Various studies have used Poly(vinylidene difluoride) (PVdF) as binder for both LIBs and SIBs^6,13,30,46^. In LIBs, PVdF is known to have good electrochemical stability, strong binding ability to both the electrode and current collector, and could absorb electrolyte that facilitates Li^+^ transport to the surface of the active materials^46–52^. However, it requires a toxic and expensive solvent (N-methyl-I-1-pyrrolidone, NMP) as its dispersing medium. Furthermore, PVdF is wettable in non-aqueous liquid electrolyte which could lead to the detachment of the electrode from the current collector, thus increasing contact resistance^46^. As for its application in SIBs, it has been reported that PVdF suffers from defluorination during sodium intercalation because of the lack of passivation in Na-ion cell^53^.

Na-carboxylmethylcellulose (CMC), a linear polymeric derivative of cellulose with different levels of carboxylmethyl substitution, is one of the aqueous binders that is considered to replace PVdF^47^. Another known water-based binder is the Styrene butadiene rubber (SBR) which possesses higher flexibility, stronger binder force, and better heat resistance than PVdF^54^. Zhang *et al*. reported that the synergistic effect of CMC/SBR offered enhanced rate capability and increased cycling stability for ZnFe_2_O_4_ anode for LIBs. Furthermore, the cross-sectional SEM images of their electrodes revealed the poor contact of the PVdF based electrode with the current collector after cycling. Also, Wang and colleagues claimed improved electrochemical performance of their MoS_2_ using CMC/SBR in comparison to PVdF^54^. It is believed that the improved electrochemical properties is due to the high tolerance of water-based polymer binders (such as CMC/SBR) against internal mechanical stress caused by volume expansion^47^. Based on these, this study focuses on the (*i*) preparation of a novel anode material FeV_2_O_4_ and (*ii*) preliminary analyses of its electrochemical properties for Na – battery systems with the incorporation of non-aqueous (PVdF) and aqueous (CMC/SBR) binders.

## Results and Discussion

**Figure 1:**
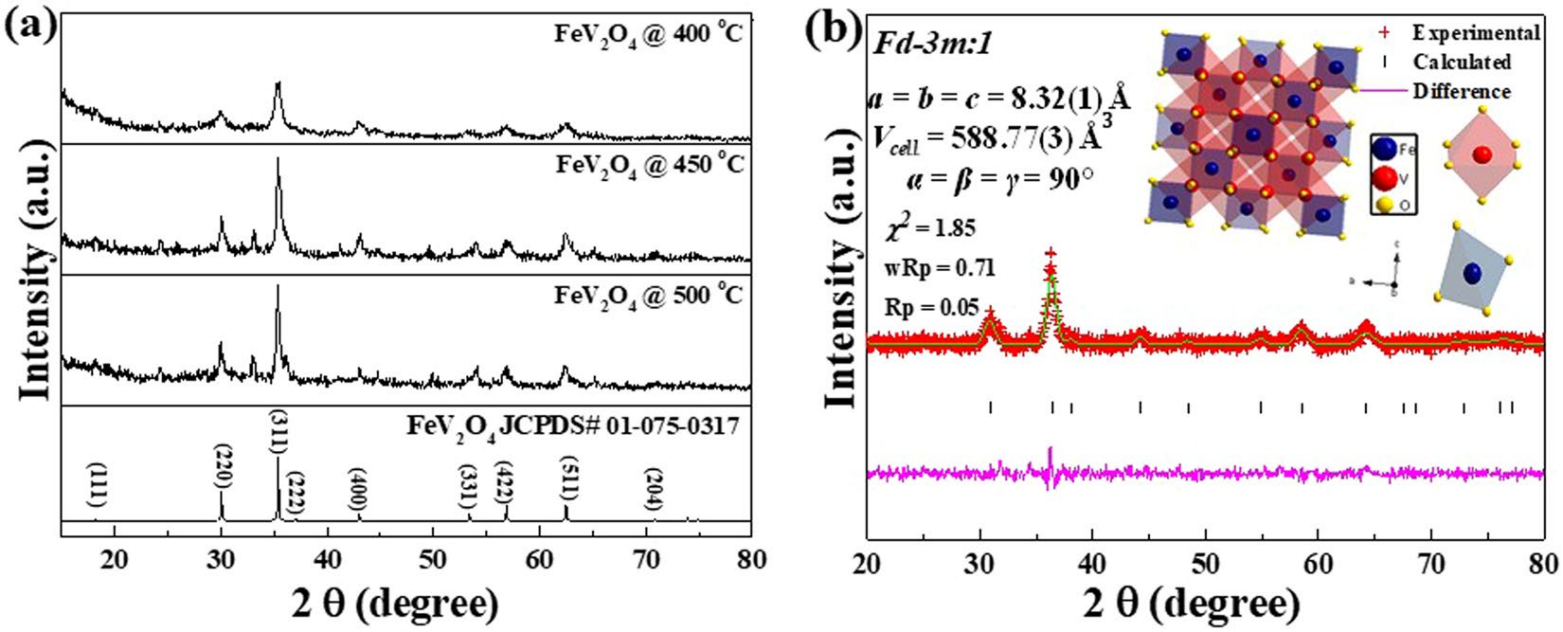
(**a**) XRD profiles of FeV_2_O_4_ calcined at different temperatures under H_2_/N_2_ atmosphere and (**b**) Rietveld refinement. Insets: crystal structure of FeV_2_O_4_ with the FeO_4_ and VO_6_ polyhedra.

The XRD profiles of FeV_2_O_4_ with different calcination temperatures (400–500 °C) are illustrated in Fig. 1(a). It was evident that the spinel structure of FeV_2_O_4_ are formed when the samples are calcined at 400 °C and all the diffraction peaks are well indexed to the standard diffraction pattern (JCPDS # 01-075-0317). Meanwhile, when the temperature was raised to 450–500 °C, impurity peaks situated at ~25°, 33° appeared which are attributable to V_2_O_3_ impurity (JCPDS # 00-001-1293). As a result, the sample calcined at 400 °C was used in the electrochemical tests. Rietveld refinement of FVO is shown in Fig. 1(b). FeV_2_O_4_ crystallizes in a face-centered cubic spinel structure with space group *Fd* $\bar{3}$*m:1*. The lattice parameters were determined to be a = 8.32(1) Å, b = 8.32(1) Å, c = 8.32(1) Å and α = β = γ = 90° with a cell volume of 588.77(3) Å^3^. The error bar of the lattice parameters and cell volume are depicted in Supplementary Fig. S1. The calculated average of the lattice parameters is 8.338 ± 0.027 Å with an average cell volume of 588.563 ± 0.291Å^3^, respectively. Lastly, the calculated crystallite size of FeV_2_O_4_ at 400 °C is 88.461 Å. The crystal structure and the corresponding coordination environments of Fe^2+^ and V^3+^ ions are displayed in the insets of Fig. 1(b). Iron cations are positioned in the tetrahedral (FeO_4_) 8a sites, while vanadium ions are situated in the octahedral (VO_6_) 16d sites and a network or grid of corner-sharing tetrahedra with cubic-closed pack oxygen anions reside in the 32e sites^55^. To further prove the crystallinity of FeV_2_O_4_ post heat-treated at 400 °C, a slower scan rate of 0.02 °∙s^−1^ was used to test its XRD. Based on Fig. S2, all peaks were indexed to the standard XRD patterns and no impurity was detected.

**Figure 2:**
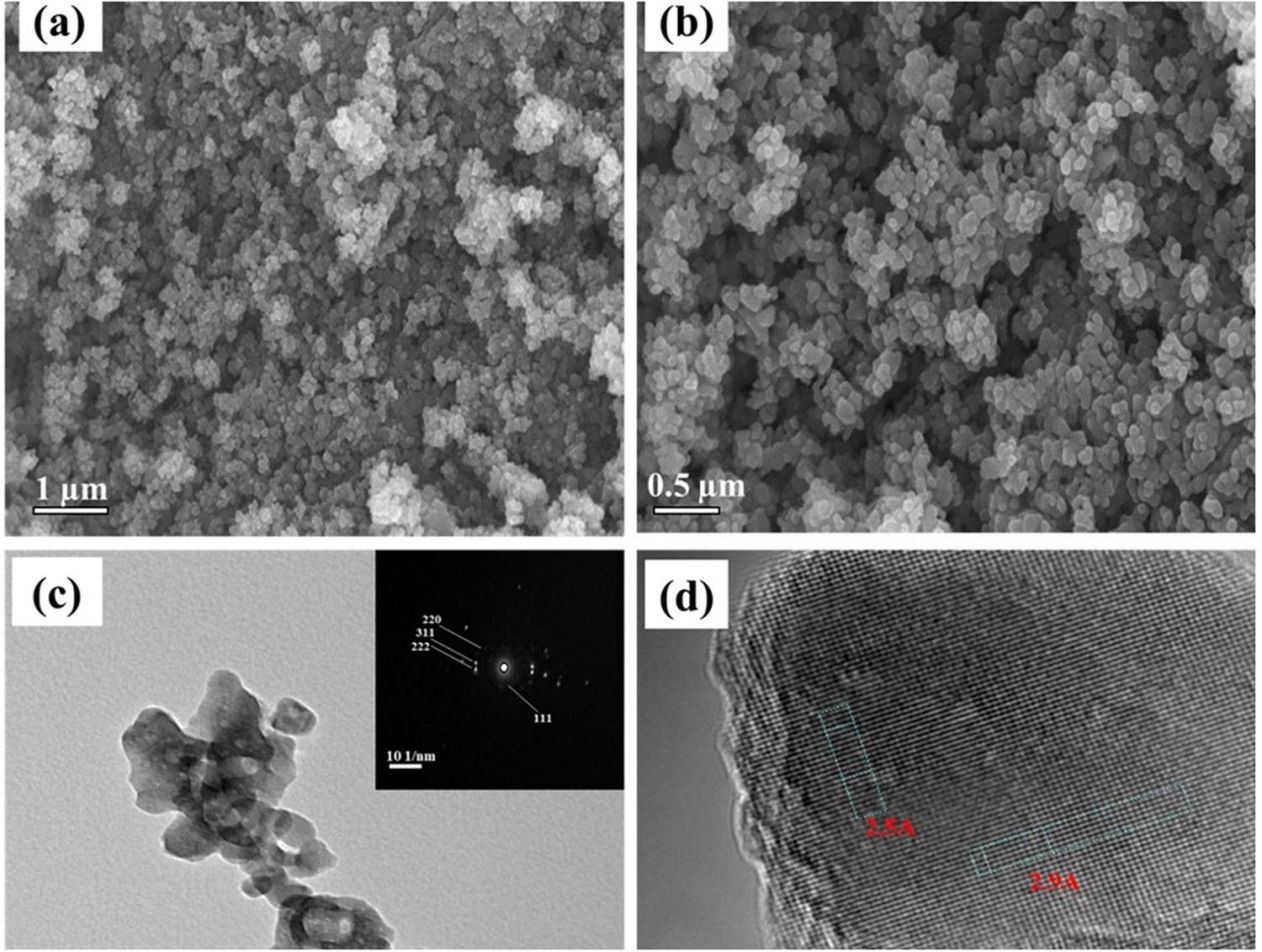
(**a**,**b**) SEM, (**c**) TEM and inset: SAED, and (**d**) HRTEM images of FeV_2_O_4_.

The morphologies and elemental compositions of FeV_2_O_4_ are depicted in Fig. 2(a–d). It was evident from the SEM images in Fig. 2(a,b) that FeV_2_O_4_ are agglomerated with irregular shapes. This is further confirmed by the TEM image in Fig. 2(c). The SAED in the inset of Fig. 2(c) confirms the crystallinity of FeV_2_O_4_ as it agrees with the characteristic diffraction peaks. HRTEM image of FeV_2_O_4_ in Fig. 2(d) show the lattice fringes of 2.9 Å and 2.5 Å which correspond to the (220) and (311) planes of FeV_2_O_4_, respectively. The elemental composition of FeV_2_O_4_ was confirmed using EDS technique as shown in Fig. S2a. The characterization was executed four times at different sites to confirm even distributions of Fe, V and O. In Fig. S2b the bar graph of the atomic % of the elements are displayed with the average compositions, corresponding standard deviations of the elements and errror bars. Based on the gathered data, the calculated At.%_AVE_ composition (Fe:V:O) = 14.35:27.3:58.35 is close to the stoichiometric ratio of FeV_2_O_4_ demonstrating uniform distribution of the elements. In addition, the elemental mapping in Fig. S3 further validates the composition and even distribution of the elements.

**Figure 3:**
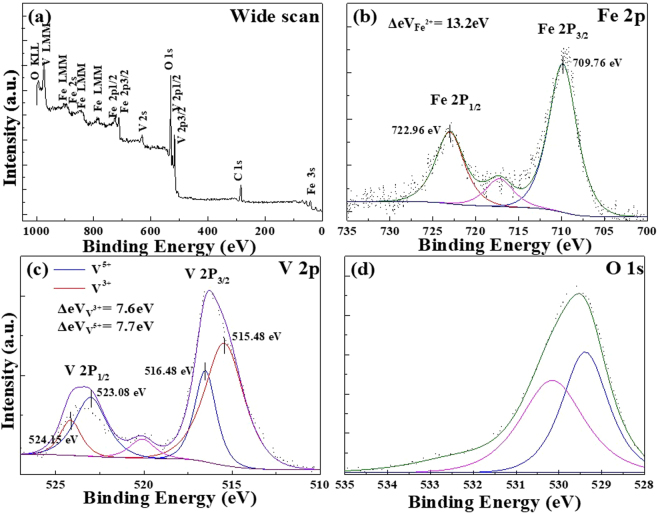
XPS of FeV_2_O_4_: (**a**) Wide scan and narrow scans of (**b**) Fe, (**c**) V, and (**c**) O.

The wide scan in Fig. 3(a) shows all the possible elements presented in the sample primarily: Iron (Fe), Vanadium (V) and Oxygen (O). Meanwhile Fig. 3(b–d) illustrates the deconvoluted narrow scans of Fe, V and O to confirm the oxidation states of each elements. Figure 3(b) reveal the existence of Fe^2+^ (FeO) as indicated by the Fe 2p_3/2_ and Fe 2p_1/2_ peaks at 709.76 eV and 722.96 eV, respectively. The pink line located at 716.5 eV was due to the satellite peak of Fe^2+^ and the spin-orbit splitting between 2p_3/2_ and 2p_1/2_ were both calculated to be 13.2 eV which concurs with the standard value of 13.1 eV. Meanwhile, both V^3+^ and V^5+^ are found to co-exist as presented in Fig. 3(c). The peaks corresponding to V 2p_3/2_ and V 2p_1/2_ are situated at 515.48 eV and 523.08 eV, respectively for V^3+^. On the other hand, V^5+^ ions have peaks situated at 516.48 eV for V 2p_3/2_ and 524.184 eV for V 2p_1/2_. The calculated spin-orbit splitting for V 2p_3/2_ and V 2p_1/2_ for both V^3+^ and V^5+^ are 7.6 eV and 7.7 eV, respectively which are in agreement with the standard value of 7.64 eV^4^. The pink line at ~ 520 eV is a satellite peak that could be attributed to the existence of both V^3+^/^5+^. The ratio of V^3+^:V^5+^ was calculated to be 3:2. The presence of V^5+^ could be attributed to slight the surface oxidation^56^. Lastly, Fig. 3d displays the XPS of O1s. The deconvoluted peaks at 530.12 eV and 529.22 eV were attributed to the metal oxides of FeO, V_2_O_3_ and V_2_O_5_.

Due to the surface oxidation of the sample, X-ray absorption near edge structure (XANES) spectrum of V K-edge were determined to probe the local oxidation of Vanadium. Supplementary Fig. S4 compares the K-edge XANES profile of V in FeV_2_O_4_ with standard metallic vanadium and vanadium oxides. The spectra in Fig. S4a display pre-edge, low-energy shoulder and edge energy absorption peaks. The pre-edge peaks correspond to the coordination environment of V in which the peak intensity is inversely proportional to the coordination geometries^57^. It is assigned as the forbidden transition 1*s* → 3*d* followed by a low-energy shoulder peak which is attributed to the 1*s* → 4*p*. Finally, the strong peak corresponds to the dipole-allowed transition 1*s* → 4*p*^57^. It can be observed that V_2_O_5_ and VO_2_ have more defined and intense peaks compared to V_2_O_3_ and FeV_2_O_4_. According to Nabavi and colleagues^58^, V^5+^ in V_2_O_5_ has both VO_4_ and VO_5_ coordination states whereas the V in standard V_2_O_3_ and FeV_2_O_4_ both occupy an octahedral site (VO_6_). Unlike V_2_O_5_, both standard V_2_O_3_ and FeV_2_O_4_ do not have a shoulder peak prior to the edge-energy absorption peak. In Fig. S4b, it can be clearly discerned that the edge energies (1*s* → 4*p)* of different vanadium oxides shift to higher energies. This energy shift is known as chemical shift which follows the Kunzl’s and is linearly proportional to the valence of the absorbing vanadium atoms^57^. The inset in Fig. S4b shows the comparison of the pre-edge and edge energy peaks of FeV_2_O_4_ with the standard V_2_O_3_ and V_2_O_5_. Although the pre-edge peaks of V_2_O_3_ and V_2_O_5_ are both located at the same peak, the intensity of V_2_O_5_ is higher because of its VO_4_ and VO_5_ polyhedra. On the other hand, the first energy edge peaks of FeV_2_O_4_ and V_2_O_3_ are close to each other. Moreover, V K-edge spectra profiles of FeV_2_O_4_ and V_2_O_3_ are qualitatively similar, thus confirming the successful synthesis of FeV_2_O_4_ with V^3+^ oxidation state.

**Figure 4:**
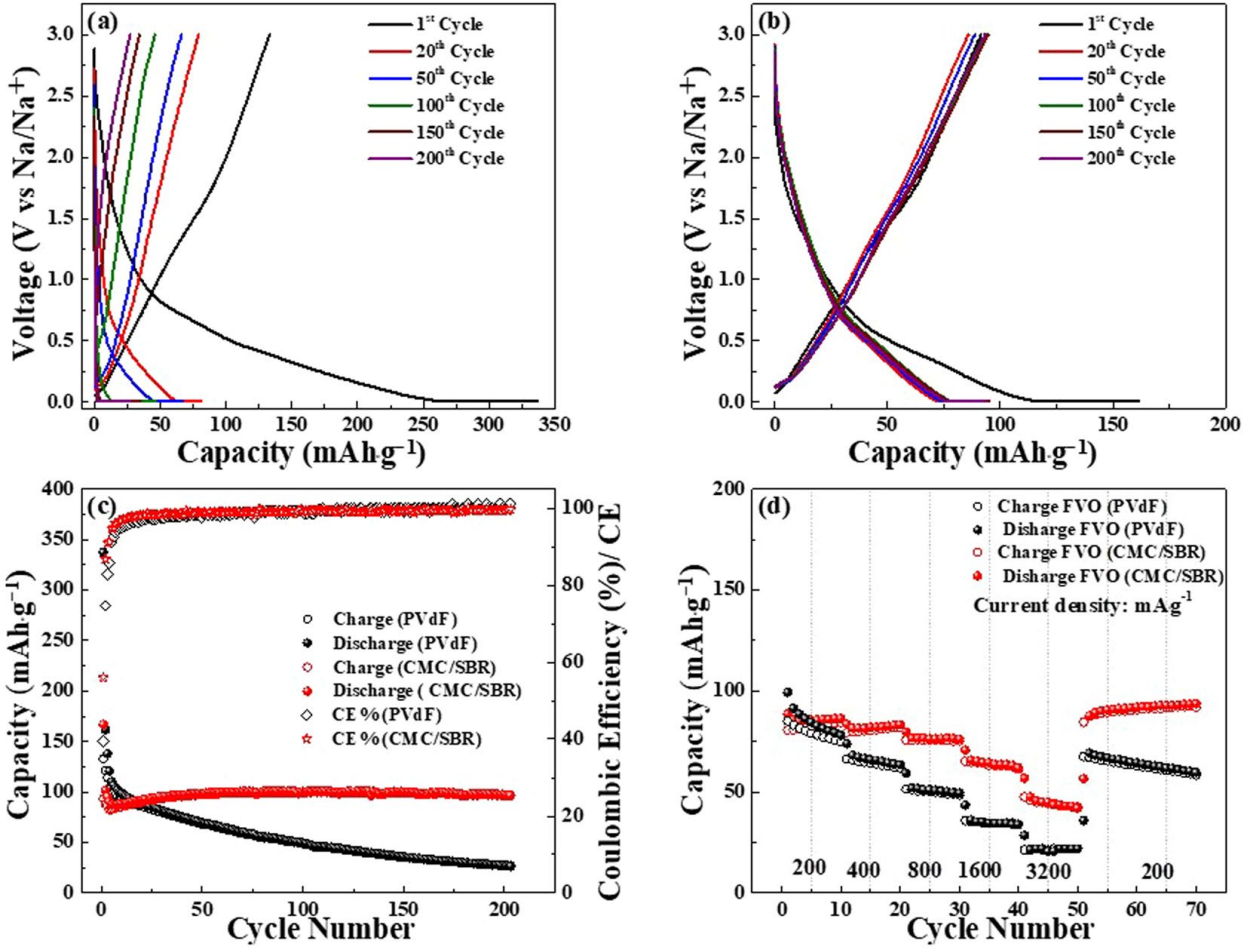
Electrochemical performance of FeV_2_O_4_ electrodes. Galvanostatic charge/discharge profiles of (**a**) FVO-PVdF and (**b**) FVO-CMC/SBR electrodes at potential window of 0.01–3.0 V. Comparisons of the (**c**) cycle life and (**d**) rate capability tests of FVO-PVdF and FVO-CMC/SBR electrodes.

The electrochemical evaluation of Na/NaClO_4_(EC:DEC)/FeV_2_O_4_ are evaluated and exhibited in Fig. 4. The galvanostatic charge/discharge of FeV_2_O_4_ using PVdF and CMC/SBR (FVO-PVdf and FVO-CMC/SBR) as binders are represented in Fig. 4(a,b). Although the intital charge/discharge profiles (at 100 mA·g^−1^) of the two electrodes look similar, the capacity of FVO-PVdF electrode was two times higher than FVO-CMC/SBR (333 mAh_∙_g^−1^ vs. 167 mAh_∙_g^−1^). However, the coulombic efficiencies were 40% and 56% for FVO-PVdF and FVO-CMC/SBR, respectively. The irreversible capacity was due to the formation of solid electrolyte interphase (SEI) layer between the anode and the electrolyte which is caused by the decomposition of the solvent in the electrolyte^14^. After prolonged cycling at 200 mA·g^−1^, the charge/discharge profiles revealed very poor stability and high voltage offset for PVdF. This was ascribed to the large polarization and mechanical energy dissipation caused by induced stress during the rapid charge and discharge process^59^. On the other hand, the profiles of the FVO-CMC/SBR electrode appear to overlap even after 200 cycles denoting good stability and low polarization.

Cycle tests with corresponding coulombic efficencies of FVO-PVdF and FVO-CMC/SBR are illustrated in Fig. 4(c). The first three cycles for formation were tested at 100 mAh_∙_g^−1^ while the rest were tested at 200 mA_∙_g^−1^. Evidently, the first few cycles of FVO-PVdF was higher than that of FVO-CMC/SBR however, prolonged cycling resulted in rapid capacity fading and lower coulombic efficiency. After 100 cycles, it was only able to retain about 39% of its capacity upon running at 200 mA_∙_g^−1^ with a coulombic efficiency of 99%. Further cycling to 200 cycles caused the battery to die eventually. It is theorized that that rapid charge/discharge caused the PVdF electrode to suffer from cracks, large volume expansion and loss of contact from the current collector. Conversely, the FVO-CMC/SBR depicts a stable cycle test upto 200 cycles. As mentioned above, the inital capacities of FVO-PVdF are higher than FVO-CMC/SBR, however, as the test continued, it was revealed that FVO-CMC/SBR delivered a more stable capacity.

It was noticeable that there was a gradual increase in the capacity, which is very common in TMOs and has been widely observed in LIBs applications. The capacity increase was due to the formation of reversible pseudo-capacitive polymeric/gel film like that is believed to be responsible for the extra uptake of Li^+^ on the SEI layer^60,61^. Since LIBs and SIBs are governed by the same rocking chair mechanism, the electrochemical properties of TMOs in SIBs are also affected by this phenomena. Moreover, since the morphology of the FeV_2_O_4_ is highly agglomerated, there are inactive sites in the electrode that didn’t form an initial reaction in the sodiation and desodiation process. With continued cycle test, nano-sized particles are believed to have formed which eventually became exposed and provided more active sites during the sodiation and desodiation process. After 200 cycles, FVO-CMC/SBR obtained a reversible capacity of ~97 mAh_∙_g^−1^ with a coulombic efficiency of 99%. At this stage, CMC/SBR was able to overcome the large volume expansion and maintained a good contact with the Cu foil. Even though FVO-CMC/SBR provided a highly stable capacity, it is obvious that the delivered capacities were very low. The theoretical capacity of FeV_2_O_4_ is believed to be ~967 mAh∙g^−1^ (1 C) considering 8 mol of Na^+^ during full conversion process. However, at 100 mA∙g^−1^ only 1.4 mol of Na^+^. This huge difference could be attributed to the follwing factors: large ionic radius of Na atom, thicker SEI layer (effect of electrolyte, which is not a scope of this research), and few active sites for Na^+^ to induce full conversion of the Fe and V metals. In order to achieve improved electrochemical properties of FeV_2_O_4_, futher developments should be done such as carbon coating to buffer volume expansion, metallic doping to increase kinetics, controlling its morphology, or providing a layered structure. And while these methods could provide increased capacity, the stability of the electrode is hugely affected by the binder.

Aside from the cycle tests, rate capability of the two electrodes were also studied and is shown in Fig. 4(d). FVO-PVdF electrode has average discharge capacities of 85, 66, 52, 36, and 22 mAh∙g^−1^ at current densities of 200, 400, 800, 1600, and 3200 mA_∙_g^−1^, respectively. When cycled back to 200 mA∙g^−1^, it was able to recover an average capacity of 63 mAh∙g^−1^, which is 25% less of the initial discharge cycle. Meanwhile, FVO-CMC/SBR electrode delivered an average discharge capacities of 86, 82, 77, 65, and 46 at 200, 400, 800, 1600, and 3200 mA∙g^−1^, respectively. Upon cycling back to 200 mA∙g^−1^, it recovered an average of 90 mAh∙g^−1^ which is higher than the former average at 200 mA∙g^−1^. These results are all indicative of the better stability that CMC/SBR offer over prolonged cycle test.

As mentioned above, it was inferred that FVO-PVdF electrode suffer from huge cracks on the surface and detachment from the Cu foil. To have further insight on the binding abilities of the different binders, *ex-situ* SEM analyses of the electrodes were performed. Supplementary Fig. S5 show the surface and cross-section morphologies of FVO-PVdF. The pristine electrode in Fig. S5a displayed uniform coating, however, it appears to have some shallow cracks which could have been formed upon drying the electrode. After the initial charge/discharge cycle (Fig. S5b), huge and deep cracks are seen on the surface and the distances between the cracks range from 1.72–7.29 µm. However, the effect of continued cycle, caused the particles to become interconnected and the cracks on the surface were lessened. Furthermore, the cross-section images were also studied as shown in Fig. S5(d–g). The pristine electrode has an average thickness (D_ave_) of 11.32 µm. After one cycle, expansion is evident, and the D_ave_ increased to 15.88 µm and the electrode was evidently detached from the current collector resulting in increased contact resistance. Further sodiation and desodiation, caused the electrode material to be severely detached from the Cu foil as shown in the low magnification image of the electrode in Fig. S5f. Also, cracks at the bottom of the electrode were present. The average thickness after 200 cycles was calculated to be 34.14 µm (Fig. S5g), indicating huge expansion by ~201%. The inevitable huge volume expansion, detachment from the current collector and cracks are attributable to the weak hydrogen bonding of fluorine in PVdF (Fig. S7a) with the active material and current collector^54,62^.

In comparison, supplementary Fig. S6 displays the *ex-situ* surface and cross-section morphologies of FVO-CMC/SBR. In Fig. S6a, the pristine electrode does not have obvious cracks on the surface and shows uniform coating. The carboxyl chains in CMC (Fig. S7b) provides an effective surface charge on the FeV_2_O_4_ and Super-P particles, therefore stabilizing the particles dispersion through an electrostatic double-layer repulsion mechanism^54,63^. After one cycle, cracks were also present on the surface as shown in Fig. S6b. However, comparing it to the cracks on FVO-PVdF electrode, the cracks appear to be shallow and short. In fact, the gaps were measured to be from 0.74–2.79 µm which are extremely smaller than that of FVO-PVdF electrode. Similarly, the surface of the electrode in Fig. S6c, appear to become denser and the particles are more connected as a result of the SEI formation on the surface and the swelling of the binder^54^. Some of the initial small pores as seen in Fig. S6a, were almost gone and the surface appeared to be smoother and more compact. In contrast to the FVO-PVdF after 200 cycles, FVO-CMC/SBR has no evident cracks on the surface. The cross-sections of the electrodes were also analyzed. The average thickness of the pristine electrode in Fig. S6d is measured to be 11.93 µm and after one charge and discharge cycle, (Fig. S6e), the D_ave_ increased to 14.04 µm. Fig. S6(f) shows the cross-section of the electrode cycled up to 200 cycles with D_ave_ = 22.40 µm, denoting 88% expansion. Huge cracks were also present, but the electrode material was still strongly attached to the Cu foil. Nevertheless, it is 2x lower than the volume expansion provided by PVdF. The relatively stronger adhesion of CMC/SBR on the current collector could be ascribed to the strong hydrogen bond of the carboxyl and hydroxyl groups in CMC with the FeV_2_O_4_, Super-P and Cu foil^54,62^. Zhang *et al*. noted that CMC makes the electrode extremely stiff and brittle when used alone as a binder. It easily forms cracks and can make the electrode slide-off the current collector. Combining CMC with SBR lessens the brittleness of the electrode. In comparison to PVdF, CMC/SBR provide smaller Young’s modulus, larger maximum elongation, and improved adhesion to the current collector^54,64^.

The plateaus in the galvanostatic charge/discharge profiles of FVO-CMC/SBR which correspond to the reduction and oxidation of Fe and V are not very distinct which is very common for other TMOs applied for both LIBs and SIBs^12,15,65–67^. Cyclic voltammetry test (CV) provides confirmation of the conversion of the metals. The CV profile of FVO-CMC/SBR is shown in Supplementary Fig. S8. It displays CV curves at a constant scan rate of 0.1 mV·s^−1^. During the first cycle, there are two broad reduction peaks located at ~0.3–0.6 V and ~1.0–1.4 V, and a narrow and broad oxidation peaks at 0.05 V and ~1.25–1.75 V, respectively. The oxidation peak at 0.05 V is attributed to Super-P. In the subsequent cycles, the reduction peak at ~0.8–1.4 V disappeared, which implies that it is due to the formation of SEI layer. The broad peak at ~0.3–0.6 V shifted to ~0.6–0.8 V in the 2^nd^-5^th^ cycles denoting irreversible electrochemical reaction in the initial discharge cycle^68^. Meanwhile, the oxidation peaks at ~1.25–1.75 V is still present in the succeeding cycles. It can be clearly seen that the CV profiles for the subsequent cycles are overlapping each other showing excellent reversibility.

It is speculated that the reduction and oxidation peaks for both Fe and V coincides with each other. In the works of Gao *et al*. on FeO/C^69^, they suggested that the reduction of of Fe^2+^  → Fe^0^ occurs at ~0.7 V and a broad oxidation peak at ~ 1.5–2.0 V that corresponds to the oxidation of Fe^0^ → Fe^2+^. Jiang and colleagues studied V_2_O_3_ nanowires for LIB and they found out that the reduction of V^3+^ → V^0^ is situated at 0.68 V and the oxidation occurs in two step process (V^0^ → V^3+^) located at 1.25 and 2.62 V^70^. The reported oxidation and reduction peaks for Fe in LIBs agree with the obtained reduction peak of Fe^2+^  → Fe^0^ at ~0.6–0.8 V, and oxidation peak of Fe^0^ → Fe^2+^ at ~1.25–1.75 V. However, taking into consideration the strong V-O bonding^34^, obtaining metallic V will be difficult. Hence, the low capacity of FeV_2_O_4_. As mentioned earlier, the CV of Li-V_2_O_3_ has a broad reduction peak (0.68 V) and two oxidation peaks (1.25 and 2.62 V). Conversely, in the CV of Na-FeV_2_O_4_, only one oxidation peak is observed. Ergo, the broad reduction peak at ~0.6–0.8 V corresponds to V^3+^  → V^2+^ and the broad oxidation peak ca. 1.25–1.75 V denotes V^2+^  → V^3+^. The CV profile of SIBs are broader than LIBs due to the larger size, heavier mass and slower mobility of Na^+^ than Li^+^^66^.

**Figure 5:**
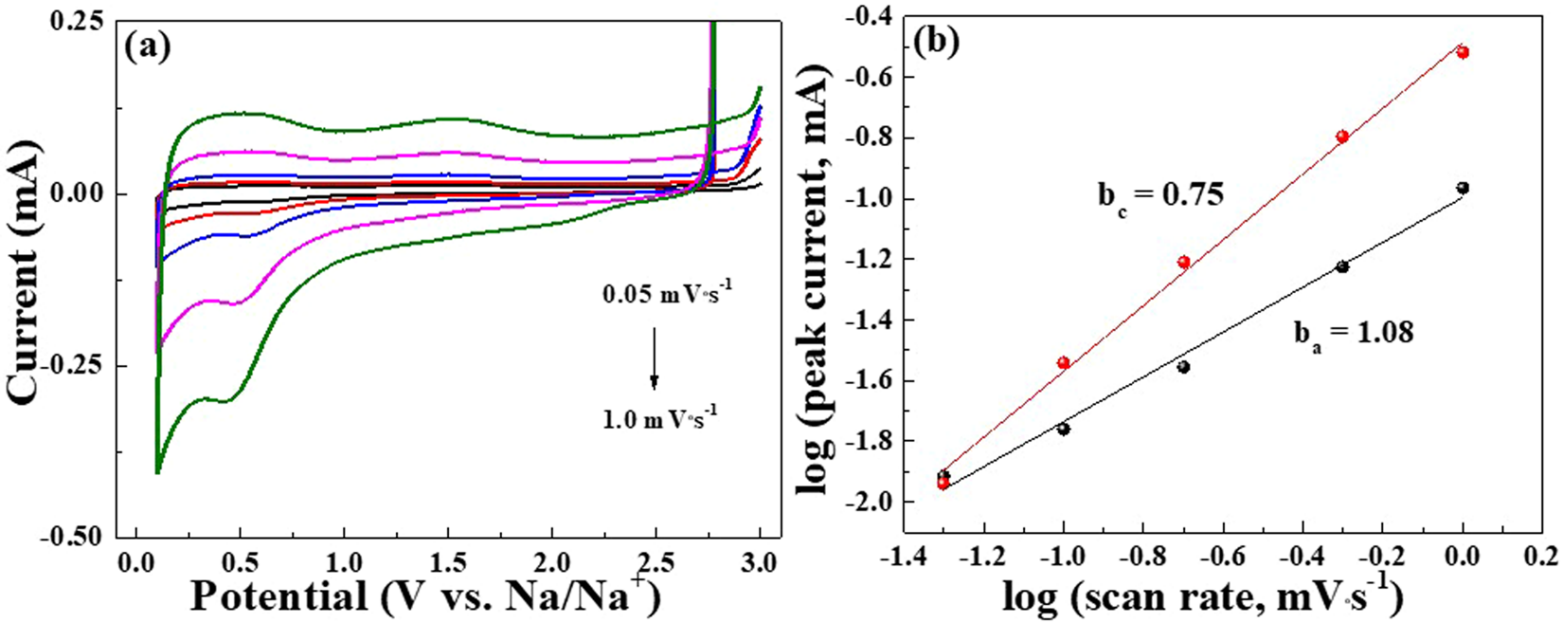
(**a**) CV curves of FVO-CMC/SBR with different scan rates from 0.05 to 1.0 mV·s^−1^ from 0.01–3.0 V. (**b**) Graph of log(*v*) vs. log(*i*).

In order to understand the capacitive behavior of FeV_2_O_4_, CV measurements at different scan rates were performed (Fig. 5(a)). As expected, the CV curves tend to deviate from its original position as the scan rate is increased which is due to the increase in polarization and ohmic resistance. The relationship between the current and the scan rate was determined using the equaiton^14^:

1 $$i=a{v}^{b}$$

where the *i* is the measured current and *v* is the scan rate. The *b*-value can be calculated using the slope of log(*v*) vs. log(*i*). If the *b*-value is close to 0.5, the electrochemical behavior is controlled by diffusion process, on the other hand, if the *b*-value is close to 1.0, it is based on capacitive process. Figure 5(b) illustrates the linear relationship of log(*v*)-log(*i*) using different scan rates and based on the fitting, the obtained cathodic and anodic *b*-value were calculated to 0.75 and 1.08, respectively. These values confirm the electrochemical behavior of FeV_2_O_4_ was mainly due to pseudo-capacitive process, hence a higly stable cycle test is obtained^14,71–73^.

To get a better understanding of the mechanism that governs conversion materials such as FeV_2_O_4_ in SIBs, it is necessary to perform *ex-situ* analyses. For TMOs, it is essential to recognize the conversion reaction that transpires during sodiation and desodiation or if there have been occurance of any phase transformations. It was mentioned that in full conversion, FVO could achieve ~967 mAh∙g^−1^, however based on the gathered data, full conversion of the metals were not obtained. Consequently, the reaction mechanism is as follows:

2 $$Fe{V}_{{2}}{O}_{{4}}+{2}xN{a}^{+}+{2}x{e}^{-}\leftrightarrow Fe+{2}VO+{2}xN{a}_{{2}}O({0} < x < {1})$$

**Figure 6:**
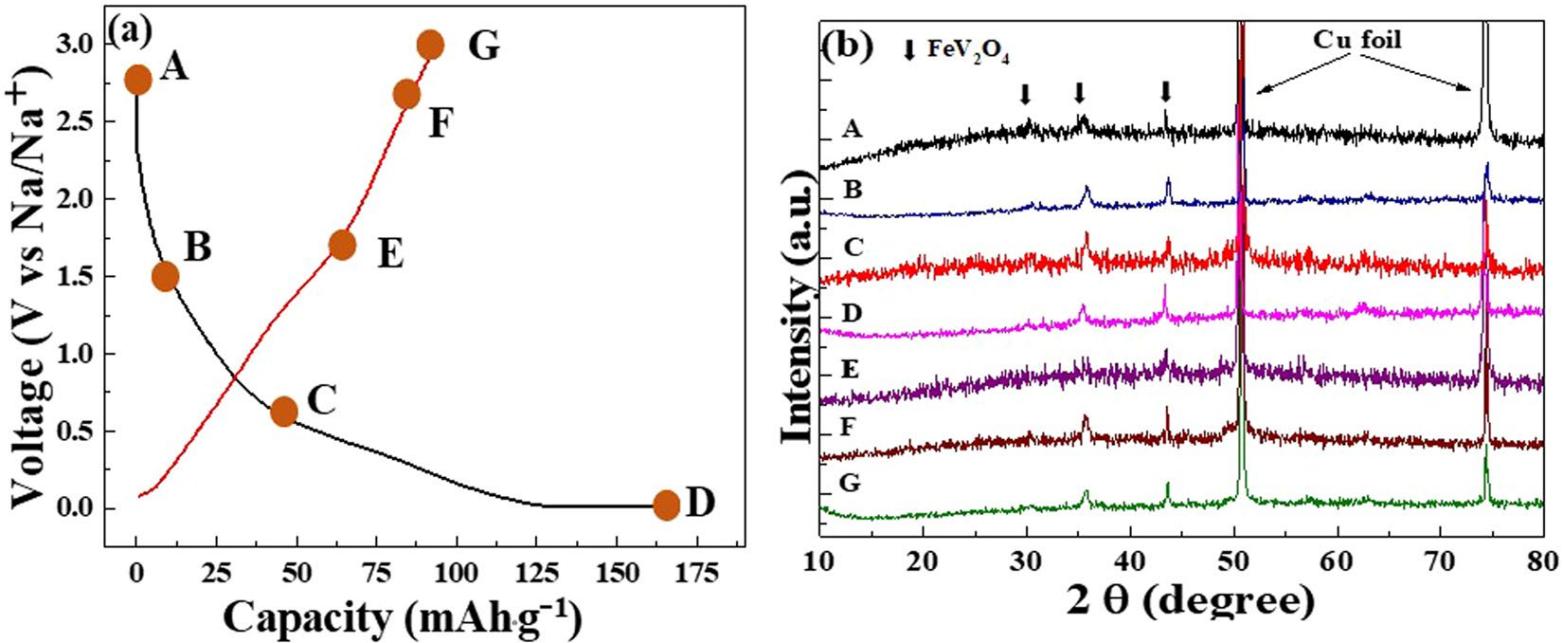
(**a**) Galvanostatic charge/discharge profile of FVO-CMC/SBR electrodes with orange marks corresponding to different depths of charge and discharge and (**b**) *Ex-situ* XRD profiles as indicated by the orange marks (A = pristine; B = D1.5 V, C = D0.5 V, D = D0.01 V and E = C1.5 V, F = C2.8 V and G = C3.0 V).

To further verify whether full conversion of the metals or any phase transformations have transpired during sodiation and desodiaion, *ex-situ* XRD characterizations of FVO-CMC/SBR electrodes were implemented. Figure 6b shows the six electrodes which were charged and discharged at certain voltages based on the CV profiles and galvanostatic charge/discharge profiles (Fig. 6(a)). The fresh electrode has the crystalline peaks of the spinel compound. When discharged and charged to different voltages, it is obvious that the XRD peaks indexed to FeV_2_O_4_ are still present indicating incomplete conversion reaction. It implies that incomplete conversion reaction occurred during sodiation and desodiation resulting in low capacities. This phenomenon is comparable to the works of Zhou *et al*.^67^ on NiFe_2_O_4_ and Mai *et al*.^74^ on NaAlTi_3_O_8_ in which their *in-situ* XRD did not indicate any conversion of the metals. However, in Fig. 6b, there is a subtle shift of the XRD peaks to the lower angle when discharged to 0.01 V. This occurance demonstrates lattice expansion due to the insertion of Na^+^ upon sodiation. Subsequently, when charged to 1.5–3.0 V, the XRD peaks indexed to FeV_2_O_4_ shifted to the right indicating release of Na^+^ upon desodiation^74^. It is also speculated that the thick SEI layer could have played a huge part on this. Hence, only few active sites were exposed to Na^+^ during sodiation.

The conversion reaction mechanism of metal oxides are not yet fully understood in Na-ion systems. Further characterizations of the electrodes using *in-situ* XRD sychrotron or *in-situ* Neutron diffraction analyses must be employed since these equipment are more highly sensitive to light elements such as Na and O^74^. Nevertheless, FeV_2_O_4_ electrode has a very high stability and reversibility which is ascribed to its pseudo-capacitive properties with the aid of the exceptional properties of CMC and SBR.

**Figure 7:**
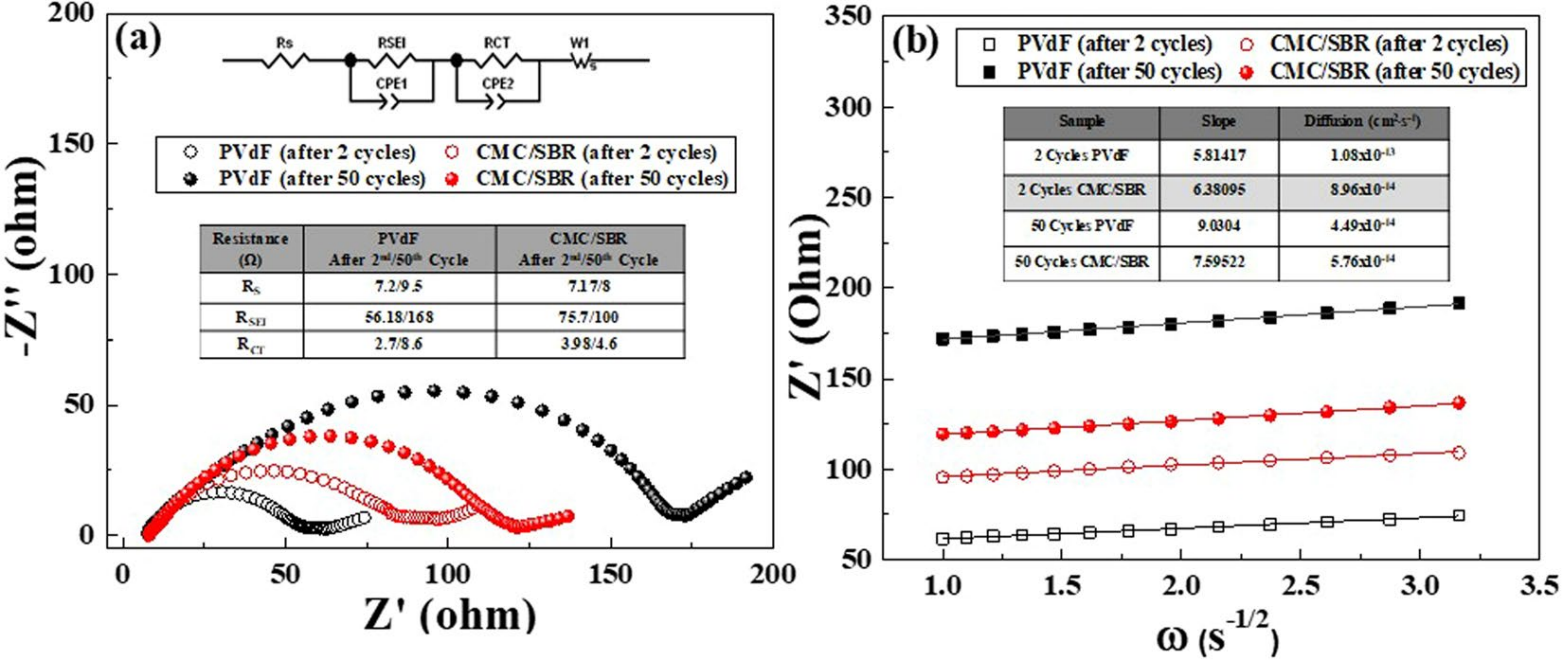
(**a**) EIS and (**b**) diffusion coefficient calculations of FVO-PVdF and -CMC/SBR electrodes.

Using Electrochemical Impedance Spectroscopy (EIS), the internal resistance in the coin cell were determined and calculated. Figure 7(a) shows a typical EIS profile which is comprised of semicircles and a straight sloping line at a lower frequency region^75^. The semicircle found in the highest frequency is denoted as R_S_ and is generally known as the electrolyte resistance. The semicircle in the middle frequency is ascribed to R_CT_ and R_SEI_ which correspond to charge transfer resistance and SEI film resistance, respectively. The sloping line, W or Warburg impedance located at the lower frequency represents the Na^+^ diffusion. The insets in Fig. 7(a) display the equivalent circuit and the calculated R_S_, R_SEI_, and R_CT_ of FVO-PVDF and -CMC/SBR after 2 and 50 cycles. The calculated R_S_, R_SEI_, and R_CT_ of FVO-PVdF electrde were 7.2, 56.18 and 2.7 Ω, after 2 cycles and increased to 9.5, 168, and 8.6, respectively after 50 cycles. On the other hand, FVO-CMC/SBR provided R_S_, R_SEI_, and R_CT_ of 7.17, 75.7 and 3.98 Ω, respectively after 2 cycles. The resistance increased to 8, 100 and 4.6 Ω for R_S_, R_SEI_, and R_CT_, respectively after the 50^th^ discharge cycle. The large R_SEI_ for both electrodes verify the poor initial coulombic efficiencies and incomplete conversion of the metals which is highly affected by the electrolyte. Comparing the two electrodes, the initial R_SEI_ and R_CT_ of FVO-PVdF is lower than FVO-CMC/SBR which is in agreement with the initial higher capacity delivered by FVO-PVdF electrode. However, after 50 cycles of (dis)charge cycles, the internal resistance of FVO-PVdF became siginificantly higher compared to FVO-CMC/SBR. These stipulate that PVdF failed to overcome the loss of contact between the active material and Cu foil and the large volume expansion that has arose during the sodiation and desodiation process hence, the large internal resistance. Moreover, it further proves that the fluorine atoms in PVdF only form weak hydrogen bonds with the active material and current collector^54^. The kinetics of the diffusion species were also investigated and calculated using the formula^76^:

3 $${\boldsymbol{\sigma }}=\frac{{\boldsymbol{RT}}}{{{\boldsymbol{n}}}^{2}{{\boldsymbol{F}}}^{2}{\boldsymbol{A}}\sqrt{2}}(\frac{1}{{{\boldsymbol{C}}}_{{\boldsymbol{Li}}}{{\boldsymbol{D}}}_{{\boldsymbol{Li}}}^{0.5}})$$

where *R* is the ideal gas constant, *T* is temperature in Kelvin, *F* is the Faraday**’**s constant, *A* is the electrode surface, *C*_*Li*_ is the concentration of Li in the electrolyte, and *D*_*Li*_ is the diffusion coefficient By calculating the slope (*σ*), from EIS, *D*_*Li*_ were calculated to be 8.965 × 10^−14^ and 5.762 × 10^−14^ cm^2^∙s^−1^ for FVO-CMC/SBR and 1.08 × 10^−13^ and 4.49 × 10^−14^ cm^2^∙s^−1^ for FVO-PVdF after the 2^nd^ and 50^th^ cycle, respectively. Initially, the Na^+^ diffusion for FVO-PVdF is slightly faster than FVO-CMC/SBR. After 50 cycles, there was a considerable decrease on the diffusion of Na^+^ ions in FVO-PVdF electrode. On the other hand, a very subtle decrease in the diffusion kinetics of FVO-CMC/SBR was observed. In fact, the increase in the internal resistance of the cell and the decrease in kinetics are very minimal even after 50 cycles. These agree with the trend in the cycle life tests in which after 50 cycles (FVO-CMC/SBR), it was able to retain 100 mAh∙g^−1^ with a very high stability suggesting that FeV_2_O_4_ could be viable conversion anode material for Na-ion battery. Although the obtained capacities were relatively low, it is believed that modifying its morphology and framework, doping with metals, and coating with carbon could highly improve its overall performance.

## Conclusions

Spinel oxide-FeV_2_O_4_ was employed as a novel anode material for sodium-ion battery. It was verified that its electrochemical behavior is mainly governed by pseudo-capacitive process. Incomplete conversion reaction was discovered in the *ex-situ* XRD. This could be due to the presence of inactive sites in the electrode, formation of thick SEI layer since no additives were used in the electrolyte and the strong V-O bonding. By using CMC and SBR as binders, a highly stable cycle test was achieved compared to PVdF. This was ascribed to the strong hydrogen bonds formed between the carboxyl/hydroxyl groups in CMC with the active material and Cu foil. Moreover, SBR provided better adhesion of the slurry to the Cu foil. Although FVO-CMC/SBR only provided a reversible capacity of ~97 mAh∙g^−1^ at 200 mA∙g^−1^ for 200 cycles, this study provided preliminary investigations on the application of FeV_2_O_4_ as a conversion based anode material for sodium-ion battery.

## Methods

### Materials synthesis

Pure-phase FeV_2_O_4_ were obtained through simple solvothermal synthesis. 1.2120 g of Iron nitrate nonahydrate (Fe(NO_3_)_2_·9H_2_O), 0.7019 g of Ammonium vanadate, (NH_4_VO_3_) were mixed in 40 mL Methanol under vigorous stirring at room temperature with the subsequent addition of 0.2521 g of Oxalic acid monohydrate. The mixture was then transferred to a 100 mL Teflon-lined stainless-steel autoclave and kept in an oven at 200 °C for 24 hours. The obtained powder was then washed repeatedly with Ethanol and Acetone and dried overnight. Finally, the precipitates were calcined at 400–500 °C for 4 hours under H_2_/N_2_ reducing atmosphere to ensure the formation of spinel compounds.

### Characterization

The crystallinity of the samples was characterized using X-ray diffractometer with Cu Kα (λ = 1.5418 Å) generated at 45 kV and 30 mA. The data were gathered in the 2θ range of 10° to 80° with a scan rate of 0.05°∙sec^−1^. XRD data were analyzed using General Structure Analysis System (GSAS) software to obtain Rietveld Refinement. The elemental compositions were analyzed through X-ray Photoelectron Spectroscopy (XPS, JEOL Photoelectron Spectrometer (ESCA), JPS-9200, monochromatic Al-Kα), V K-edge X-ray absorption spectra (XAS conducted at National Synchrotron Radiation Research Center (NSRRC), Hsinchu, Taiwan using BL01C1 and BL17C1 beamlines) and Energy Dispersive X-ray Spectroscopy (EDS, X-MAX). The morphology and elemental mapping and crystal structure of the samples were analyzed via tunneling electron microscope Cs-corrected Scanning Transmission Electron Microscope (FEI-Titan3 G2-60-300 operating at 200 kV and scanning electron microscope (SEM) – Hitachi S-4100. For *ex-situ* XRD characterizations, the batteries were opened after designated charge and discharge voltages inside an Ar-gas filled glove box with with H_2_O and O_2_ content <0.5 ppm. The anode electrodes were carefully collected, washed with dimethyl carbonate (DMC) to remove the electrolyte and were vacuumed overnight to remove excess solvents.

### Electrochemical Measurement

The electrochemical performances of the batteries were measured by assembling CR2032 coin cells. Two slurries were prepared with similar compositions of 70:15:15 for active material (FeV_2_O_4_), Super-P (Carbon black, 40 nm), and binder. For aqueous based binder, 9 wt.% of CMC (Mw = 2 × 10^5^ Da) and 6 wt. % of SBR was dissolved in DI H_2_O. Meanwhile, 15 wt. % of PVdF (Mw = 1 × 10^6^ Da) was dissolved in NMP. The prepared slurries were coated onto 10 μm copper foil which was used as the working electrode of the battery. The samples were punched (14 mm) and dried at 120 °C for 8 h in vacuum system to remove the residual solvents. The batteries were assembled in an Ar-gas filled glove box with H_2_O and O_2_ content <0.5 ppm using sodium disks as the counter electrode, 1 M of NaClO_4_ in ethylene carbonate (EC) and diethyl carbonate (DEC) (1:1 in volume ratio) as electrolyte, and glass fiber filter disks as the separators. The discharge/charge tests were analysed using AcuTech System in the voltage range of 0.01 V and 3.0 V at room temperature constant voltage charge process. The mass loading of these sample is in the range of 2.60 ± 0.30 mg/cm^2^. The cyclic voltammograms (CV) were measured by CH Instruments Analyzer CHI 6273E at a scan rate of 0.1 mV·s^−1^ between 0.01 V and 3.0 V and the Electrochemical Impedance of the samples were tested in the frequency range from 0.01-100000 Hz. For the *ex-situ* analyses, the electrodes were opened inside an Ar-gas filled glove box with H_2_O and O_2_ content <0.5 ppm. The electrodes were washed with dimethyl carbonate (DMC) to remove excess electrolytes and were dried inside the vacuum chamber overnight to prevent oxidation.

## Electronic supplementary material

Supplementary Information
