# Supplementary material for: Electrochemical properties of novel FeV2O4 as an anode for Na-ion batteries
Source: Sci Rep. 2018 Jun 11;8:8839. doi: 10.1038/s41598-018-27083-z (PMC5995833; doi:10.1038/s41598-018-27083-z)
Supplement: Supplementary file 1 — Supplementary Information [file 41598_2018_27083_MOESM1_ESM.docx]

**Supporting Information**

**Electrochemical properties of novel FeV_2_O_4_ as an anode for Na-ion batteries**

**Irish Valerie B. Maggay,^1†^ Lyn Marie Z. De Juan,^2†^ Jeng-Shin Lu,^1†^ Mai Thanh Nguyen,^2^ Tetsu Yonezawa,^2^ Ting-Shan Chan,^3^ and Wei-Ren Liu^1*^**

^1^Department of Chemical Engineering, Chung Yuan Christian University, Taoyuan City, Chungli 32023, Taiwan, R.O.C.

*E-mail: [WRLiu1203@gmail.com](mailto:WRLiu1203@gmail.com)

Tel:+886 3-265-4140; fax: +886 3-265-4199

^2^Division of Materials Science and Engineering, Faculty of Engineering, Hokkaido University

Kita 13 Nishi 8, Kita-ku, Sapporo, Hokkaido 060-8628, Japan

^3^National Synchrotron Radiation Research Center (NSRRC), Hsinchu, 30076, Hsinchu, Taiwan

Fig. S1. (a) Lattice parameters and (b) cell volume of FeV_2_O_4_ with their corresponding error bars.

Fig. S2. Comparison of FeV_2_O_4_ calcined at 400°C with different XRD scan rate.


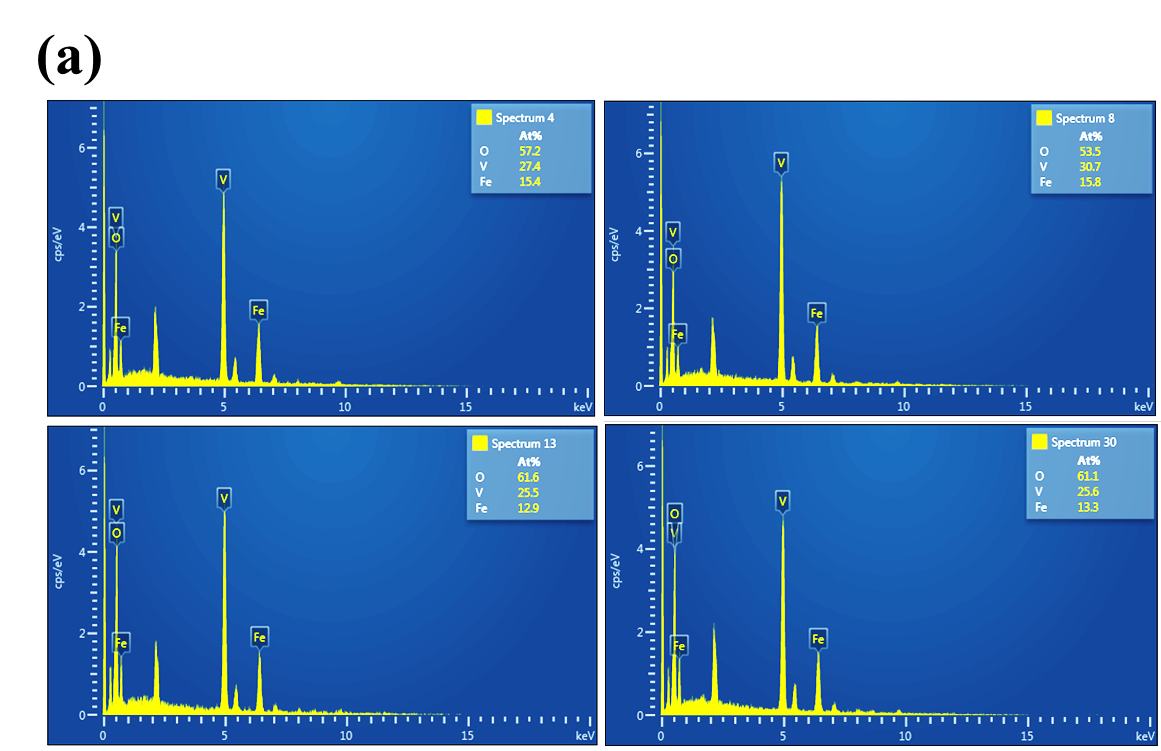

Fig. S2 (a) EDS spectra of FeV_2_O_4_ and (b) its error bar.


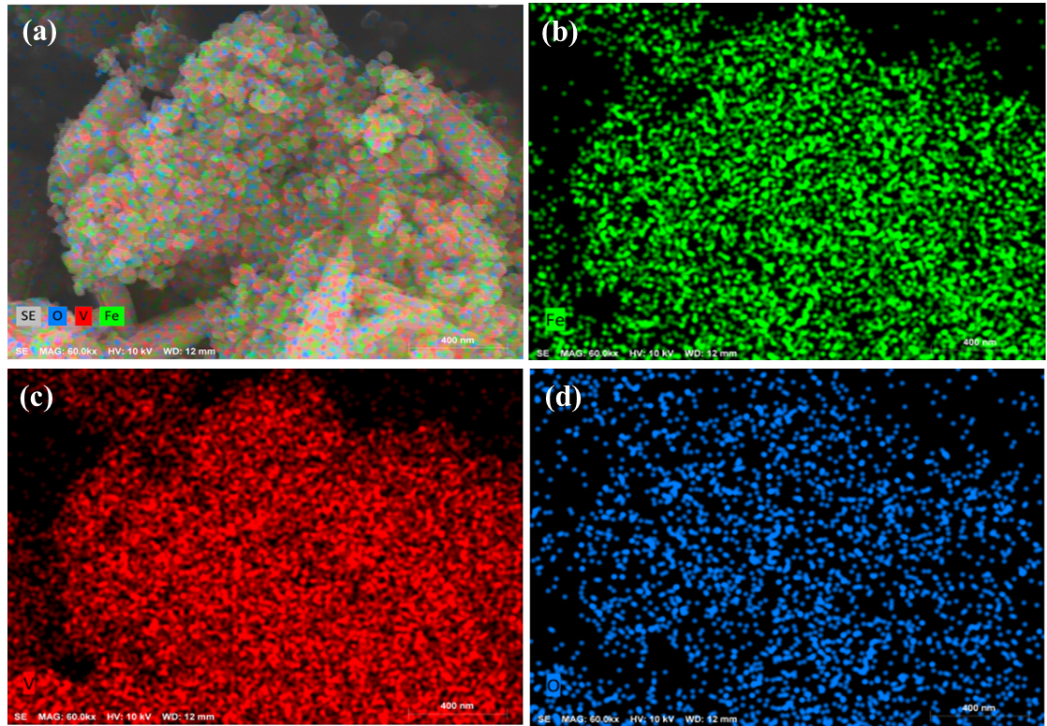


Fig. S3. Elemental mapping of FeV_2_O_4_.

Fig. S4. (a) X-ray absorption near edge (XANES) spectra of V K-edge and the (b) zoomed in graph of the edge energies.


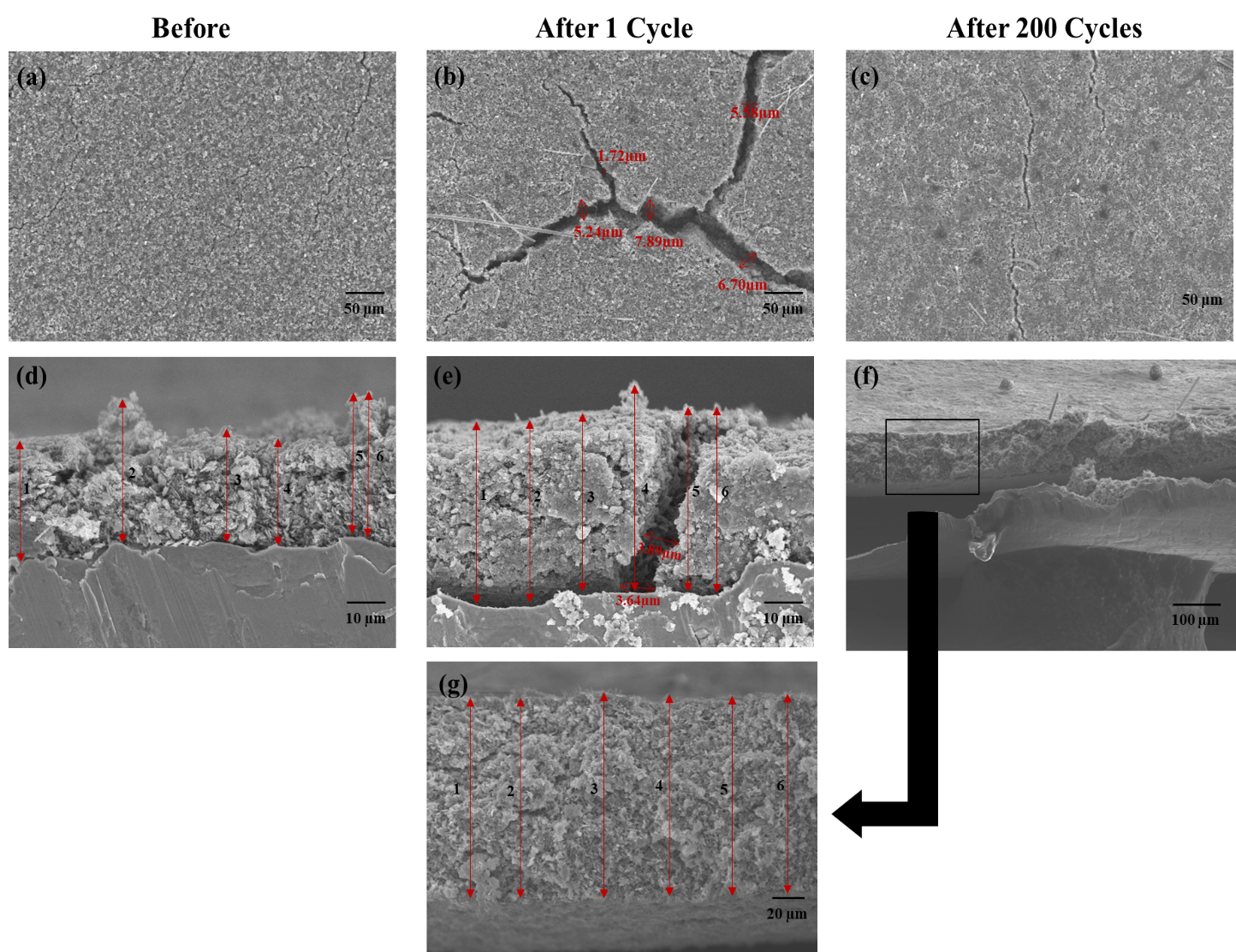


Fig. S5. Ex-situ SEM images of FVO-PVdF electrodes (a) before, after the (b) 1^st^ and (c) 200^th^ charge/discharge cycle. Cross-section SEM images of (d) before, (e) after the 1^st^_,_ and (f) low and (g) high magnification of the electrode after the 200^th^ cycle.

Table 1. Measured thickness of FVO-PVdF electrode before and after charge/discharge cycle.

| FVO-PVdF | Before  (µm) | After 1^st^ C/D Cycle (µm) | After 200^th^ C/D Cycle (µm) |
| --- | --- | --- | --- |
| D_1_ | 11.00 | 15.82 | 34.64 |
| D_2_ | 13.08 | 15.03 | 34.43 |
| D_3_ | 9.14 | 15.36 | 34.65 |
| D_4_ | 9.93 | 17.66 | 34.10 |
| D_5_ | 12.34 | 15.76 | 33.44 |
| D_6_ | 12.45 | 15.70 | 33.55 |
| D_ave_ | **11.32** | **15.88** | **34.14** |
| **Expansion: 201%** | | | |


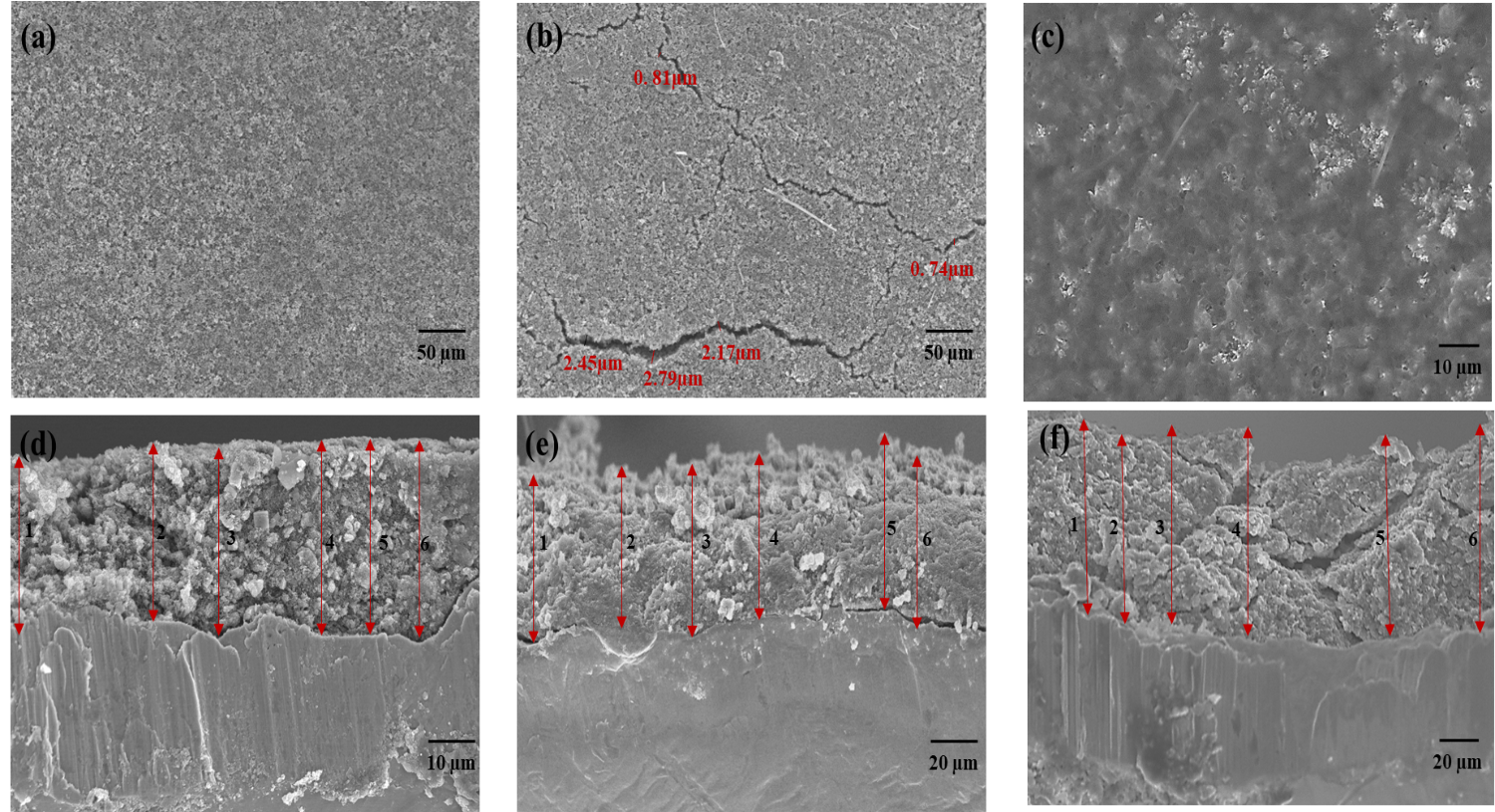


Fig. S6. Ex-situ SEM images of FVO-CMC/SBR electrodes (a) before, after the (b) 1^st^ and (c) 200^th^ charge/discharge cycle. Cross-section SEM images of (d) before, after the (e) 1^st^ and (f) 200^th^ charge/discharge cycle.

Table 2. Measured thickness of FVO-CMC/SBR electrode before and after charge/discharge cycle.

| FVO-CMC/SBR | Before  (µm) | After 1^st^ C/D Cycle (µm) | After 200^th^ C/D Cycle (µm) |
| --- | --- | --- | --- |
| D_1_ | 11.07 | 13.72 | 21.46 |
| D_2_ | 11.22 | 13.43 | 21.45 |
| D_3_ | 12.11 | 14.35 | 22.96 |
| D_4_ | 12.31 | 13.72 | 23.49 |
| D_5_ | 12.12 | 14.56 | 22.51 |
| D_6_ | 12.73 | 14.44 | 22.53 |
| D_ave_ | **11.93** | **14.04** | **22.40** |
| **Expansion: 88%** | | | |

**(a)**

**(b)**

**(c)**

Fig. S7. Molecular structures of (a) PVdF, (b) CMC^1^, and (c) SBR.

1 Biswal, D. & Singh, R. Characterisation of carboxymethyl cellulose and polyacrylamide graft copolymer. *Carbohydr. Polym.* **57**, 379-387 (2004).

Fig. S8. CV profiles of FVO-CMC/SBR at a scan rate of 0.1 mV·s^-1^ from 0.01 – 3.0V.
